# Motivation and perceived competence for healthy eating and exercise among overweight/obese adolescents in comparison to normal weight adolescents

**DOI:** 10.1186/s40608-017-0172-2

**Published:** 2017-11-17

**Authors:** Suzanne Mokhtari, Benjamin Grace, Youngju Pak, Astrid Reina, Quinn Durand, Jennifer K. Yee

**Affiliations:** 1 Department of Pediatrics, Division of Hospital Medicine, Los Angeles Biomedical Research Institute at Harbor-UCLA Medical Center, 1124 W. Carson Street, Torrance, CA USA; 2 Department of Pediatrics, Division of Pediatric Endocrinology, Los Angeles Biomedical Research Institute at Harbor-UCLA Medical Center, 1124 W. Carson Street, Torrance, CA USA; 3 Department of Medicine, Los Angeles Biomedical Research Institute at Harbor-UCLA Medical Center, 1124 W. Carson Street, Torrance, CA USA; 4 Department of Psychiatry, Division of Psychology, Los Angeles Biomedical Research Institute at Harbor-UCLA Medical Center, 1124 W. Carson Street, Torrance, CA USA

**Keywords:** Obesity, Adolescent, Motivation, Self-efficacy, Perceived competence

## Abstract

**Background:**

The current literature on determinants of behavior change in weight management lacks sufficient studies on type of motivation among children/adolescents, on perceived competence, and in relation to healthy eating. This study aimed to investigate type of motivation and levels of perceived competence for healthy diet and exercise, as well as general self efficacy among adolescents. We hypothesized that overweight/obese adolescents would demonstrate lower autonomous motivation and perceived competence regarding diet and exercise, and lower self-efficacy in general, and that the scores would be influenced by socioeconomic factors.

**Methods:**

Normal weight (*n* = 40, body mass index < 85% for age and gender) and overweight or obese adolescents (*n* = 60, body mass index ≥ 85% for age and gender) aged 13-18 years were recruited from pediatric ambulatory clinics. Information was collected about demographics, socioeconomic factors, and lifestyle behaviors. The study subjects completed a survey including the Treatment Self-Regulation Questionnaire (TSRQ) and the Perceived Competence Scale (PCS) for healthy eating and exercise, and the General Self-Efficacy Scale (GSES). Composite scores for the three scales were compared between the two groups using the using the two-sample t-test (for normal data) or the Mann-Whitney U test (for non-parametric data). Relationships between the composite scores and patient characteristics were determined using Pearson or Spearman’s correlations.

**Results:**

The average age of the total cohort was 15.9 ± 1.9 years. 54% were female, and 82% identified as Latino/Hispanic. In comparison to normal weight subjects, overweight/obese adolescents exhibited higher scores for controlled motivation (mean ± standard deviation 28.3 ± 9.3 vs 18.1 ± 8.1) and higher perceived competence [median and 25-75% interquartile range 22.5 (19.0-26.0) vs 20.0 (15.5-25.0)] in relation to eating a healthy diet. These differences persisted after adjustment for age, sex, paternal education, and family income.

**Conclusions:**

Overweight/obese adolescents did not lack autonomous motivation but demonstrated higher controlled motivation and perceived competence for healthy eating in comparison to normal weight adolescents, independent of socioeconomic factors. In the clinical practice of weight management, providers should carefully assess adolescents for type of motivation and perceived competence, while accounting for potential barriers to behavior change.

## Background

Overweight and obesity affect 32% of children and 69% of adults in the United States [1]. Health disparities in obesity exist, with Hispanic, Black, and low-income children affected at disproportionately higher rates [2–4]. Lifestyle modification remains the cornerstone for weight management interventions [5]. Successful weight loss and maintenance are dependent upon behavior change and sustaining healthy behaviors over time [6, 7].

Many studies in obesity address motivation among other determinants of behavior change. Most of these studies were conducted among adults [6–9], with results indicating that higher motivation is associated with behaviors related to weight control. However, children and adolescents are less mature than adults in social and cognitive development, including frontal lobe functions such as impulse control, attention span, and motivation [10, 11]. The majority of the few pediatric studies focus on physical activity, with results supporting an association between higher motivation and higher physical activity [12]. One study of adolescents in Korea [13] suggested that motivation for physical activity is lower in obese children. Application of the current evidence to pediatric obesity clinical care is challenging due to the highly diverse nature of the literature, with variations in subject age, settings, terminology used for psychological constructs, psychometric measures, and behaviors studied (more exercise studies, fewer on healthy eating). Additional studies are needed to establish baselines across different populations, address type of motivation among children, and in relation to healthy eating.

Motivation is a determinant based on the Self-Determination Theory (SDT) [14], which distinguishes between autonomous motivation, controlled motivation, and amotivation. Autonomous motivation originates from one’s self, encompassing a personal belief that a behavior is important. In contrast, controlled motivation results from external influences such as peer pressure or environmental circumstances. Amotivation describes an individual’s lack of any compelling reason for a behavior. In the clinical setting, a better understanding of motivational factors could potentially assist physicians who are aiming to promote healthy behaviors in their patients [15–17]. Motivation-based interventions, in complement to SDT, can then be employed to promote behavior change, with the potential to lead to weight loss [18].

Perceived competence is another component of the SDT, referring to the self-perception that one can accomplish a challenging task and be successful in meeting goals, such as those related to healthy eating and exercise. Self-efficacy is a complementary concept based on the social-cognitive theory and refers to one’s confidence in the ability to carry out a behavior even when faced with challenges. While these terms are often casually interchanged, they are addressed separately in this project. Both constructs are believed to predict, if not precede, behavior change. Studies on adults [19–21], and to a lesser extent on youths [22], suggest an association between self-efficacy and self-regulation of eating behaviors. The limited existing literature on perceived competence among adolescents does not address obesity [23]. In clinical evaluation and management of childhood obesity, perceived competence for healthy behaviors and self-efficacy may increase the potential for behavior change to promote weight control, and therefore requires further study.

Los Angeles includes many communities who experience high rates of obesity [24] and obesity-related complications. Socioeconomic status (SES) has been shown to influence obesity [25], specifically in relation to income [26], parental education [27], and adolescent education [28]. However, it is not clear how SES factors impact motivation, perceived competence, and self-efficacy in relation to weight-related behaviors. A baseline understanding of factors contributing toward adolescent behavior in a patient population is important to establish when planning for implementation of behavioral health interventions. The primary aim of the present study was to determine type of motivation and perceived competence for healthy diet and for physical activity, along with general self-efficacy, among adolescents from pediatric clinics in a publicly-funded health care system. The secondary aim was to determine whether any socioeconomic factors influenced the outcomes. Finally, we explored any potential relationships between our primary outcomes and self-reported health behaviors. We hypothesized that overweight and obese adolescents would demonstrate lower autonomous motivation, perceived competence for healthy diet and exercise, and self-efficacy in general, and that these constructs would be influenced by socioeconomic factors.

## Methods

### Study design and subject recruitment

The study was approved by the Human Subjects Committee at the Los Angeles Biomedical Research Institute at Harbor-University of California, Los Angeles (Harbor-UCLA). In this study, normal weight and overweight/obese adolescents were recruited, and the two groups were compared.

Subjects were recruited from the pediatric ambulatory clinics at Harbor-UCLA Medical Center, which is part of a publicly-funded health care system in greater Los Angeles. The majority of patients have government-sponsored health plans. Patients aged 13-18 years were approached and screened for eligibility prior to or following scheduled appointments. Subjects were excluded for pregnancy, developmental delay/ mental retardation, acute illness, or acute worsening of a chronic disease, since these characteristics might unpredictably affect psychometric measures. Subjects with stable chronic conditions were included, as the project aimed to better understand this health care network’s patient population which includes patients with chronic conditions. Research team members presented the study as an investigation of adolescents’ motivation to adopt or maintain a healthy lifestyle. The study was not presented as an obesity or weight-related research project.

If eligible, the parents of subjects age 13-17 provided informed consent, and the child provided assent, while subjects who were 18 years old provided informed consent. Information was collected about demographics, medical history, social factors, and lifestyle habits such as daily hours spent doing physical activity, number of sodas and juices consumed per day, number of meals eaten per week at home vs. restaurants, and hours of screen time.

From chart review, height, weight, and blood pressure were recorded, and past medical history was reviewed. All vital sign measurements were performed by clinic nursing staff as standard of care. For compensation, subjects received a gift pack promoting healthy living (Frisbee, low calorie snacks, and educational materials). The body mass index (BMI) percentiles and z-scores were calculated based on the Centers for Disease Control growth charts from 2000 [29].

The subjects were asked to complete the following scales which were previously published and validated: 1) the Treatment Self-Regulation Questionnaire (TSRQ), 2) the Perceived Competence Scale (PCS), and 3) the General Self-Efficacy Scale (GSES). The TSRQ for eating a healthy diet and exercising regularly included 15 items pertaining to autonomous, controlled, and amotivational factors [30]. Each of the 15 items began with the statement: “The reason I would eat a healthy diet (or exercise regularly) is…” An example of one item was: “Because I feel that I want to take responsibility for my own health.” Participants were asked to rate how true each statement was using a 7-point scale. The PCS separately addressed eating healthy and exercising using 4 items each [31]. Participants were again asked to rate how true a statement was using a 7-point scale. An example of one PCS item was: “I feel confident in my ability to maintain a healthy diet.” For the TSRQ and PCS scales, study subjects interpreted healthy eating and exercising regularly according to their own subjective definitions, and responses are assumed to be based accordingly. The GSES is a 10-item scale used to predict coping with a variety of life difficulties, but the items do not specifically relate to diet or exercise behaviors [32]. Subjects were asked to rate how true each of the statements was for them using a 4-point scale. An example of a GSES item was: “I can always manage to solve difficult problems if I try hard enough.”

In the TSRQ, composite scores were calculated by adding the scores for the individual items belonging to each type of motivation, thus creating a continuous variable. For the PCS, separate composite scores were calculated for healthy eating and exercise, as the sum of all items in each scale. The GSES composite score was also calculated as the sum of all items. If individual survey items from the questionnaires were left blank by any study subject, the subject’s composite score was excluded from analysis.

### Statistical analysis

One hundred total subjects were recruited. In study design, a power calculation was made based on available data in the literature on general self-efficacy scores. A total sample size of 100 (with *n* = 40 in one group), was anticipated to have 80% power to detect the effect size of 0.6 in a two group comparison when the mean self-efficacy score of the normal weight group and the standard deviation are assumed to be 27 and 5 [33], respectively, with the level of significance of 0.05.

Based on BMI percentile scores, the subjects were determined to be either overweight (BMI ≥ 85% for age and gender), obese (BMI ≥95% for age and gender), or normal weight (BMI < 85% for age and gender). The overweight and obese subjects were included in one group for the following reasons: 1) Clinically, the overweight subjects were more similar to the obese subjects with higher blood pressures, and therefore experience the same screening and interventions from health care providers as obese adolescents do. 2) In the literature, health-related quality of life is affected in both overweight and obese youth compared to a reference population [34].

The primary outcomes were the composite scores for the categories of motivation in the TSRQ, PCS, and GSES scales. Secondary outcomes included patient, family and socioeconomic characteristics. Exploratory outcomes included self-reported behaviors including screen time, dietary, and physical activity behaviors.

SYSTAT and SigmaStat (Systat Software, San Jose, CA) were used for statistical analysis. Distribution plots of the variables were examined to qualitatively assess for normality in addition to Shapiro-Wilk testing. For the psychometric measures, Cronbach’s alpha was computed to determine internal validity. For the primary aim, composite scores for psychometric measures were compared between the normal weight and overweight/obese groups using the two-sample t-test for normal data, or Mann-Whitney U test for non-normal data. To determine whether patient characteristics (e.g. age) or socioeconomic traits that were found to be different contributed to score variation (secondary aim), Pearson or Spearman’s correlations analyzed the relationship between the primary outcome measures and those specific traits, then analyses of covariance (ANCOVA) were performed to determine the effect of BMI status (factor) and these potential confounders on scores. Statistical significance was set at *p* < 0.05. Exploratory analyses by Pearson or Spearman’s correlations were performed to determine potential relationships between the composite scores and self-reported behaviors.

## Results

### Subject characteristics

The characteristics of the study participants are summarized in Table 1. One hundred subjects enrolled in the study. One hundred four individuals signed for consent and four dropped out of participation. The response rate was ≥90% for each characteristic, except for family income (85%). For the entire cohort, the average age was 15.9 ± 1.9 years and 54% were female. 82% of subjects identified as Latino/Hispanic origin. The average BMI was 27.3 ± 7.8 kg/m2 (z-score of 1.1 ± 1.3), with 60% having BMI ≥ 85% for age and gender. The parents on average achieved a 9th grade education and the median family income was ~$20,000 per year.

**Table 1:** Characteristics of study participants

	Total cohort	Normal weight (BMI < 85%)	Overweight/Obese (BMI ≥ 85%)
Number of subjects, n	100	40	60
Age, years	15.9 ± 1.9	16.0 ± 1.8	15.8 ± 1.9
Gender, Male/Female	46 M/54F	20 M/20F	26 M/34F
Ethnicity, N (%)	Latino/Hispanic, 82 (82%)	Latino/Hispanic, 32 (80%)	Latino/Hispanic, 50 (83.3%)
	Black/African American, 4 (4%)	Black/African American, 2 (5%)	Black/African American, 2 (3.3%)
	Non-Hispanic White, 8 (8%)	Non-Hispanic White, 3 (7.5%)	Non-Hispanic White, 5 (8.3%)
	Asian, 3 (3%)	Asian, 2 (5%)	Asian, 1 (1.7%)
	Other, 3 (3%)	Other, 1 (2.5%)	Other, 2 (3.3%)
Grade Level in School	10.1 ± 1.8	10.2 ± 1.9	10.1 ± 1.8
Mother’s highest education level	9.5 ± 4.1	9.2 ± 4.6	9.7 ± 3.8
Father’s highest education level	9.7 ± 4.0	10.7 ± 4.2	9.1 ± 3.8*
Annual family Income ($) (*n* = 85)	20,800 (15,540-28,500)	25,000 (16,200-43,750)	18,000 (15,000-23,000)*
BMI, kg/m2	27.3 ± 7.8	20.2 ± 2.9	32.0 ± 6.5*
BMI z-score	1.1 ± 1.3	−0.26 ± 1.1	1.94 ± 0.5*
Blood Pressure Systolic, mmHg	119 ± 11.3	111 ± 10.3	124 ± 8.8*
Blood Pressure Diastolic, mmHg	66 ± 7.7	63 ± 7.4	67 ± 7.4*

Legend: *N* = 100 in the total cohort. The subjects were included in the Normal weight category if the body mass index (BMI) was less than 85% for age and gender, or in the Overweight/Obese category if the BMI were greater than or equal to the 85% for age and gender. The data are expressed as the means ± standard deviation (SD) or median (25-75% interquartile range)

*p < 0.05 for comparisons between normal weight subjects and overweight/obese subjects

In comparison of overweight/obese subjects to normal weight subjects, there were no differences in age, distribution of ethnicity, or mother’s education level. The overweight/obese group exhibited lower paternal education level and annual family income. Overweight/obese subjects demonstrated higher blood pressures.

### Test responses and internal validity

The test completion rate was above 95% for all tests except GSES, which had a 77% completion rate. Of note, the GSES was the last test on the questionnaire. Subjects that did *not* complete the GSES differed from those who completed the test in three characteristics: 1) lower grade level in school (9.3 ± 1.7. vs. 10.4 ± 1.8), 2) lower maternal education level (7.8 ± 3.6 vs. 10.0 ± 4.2), and 3) lower estimated family income ($16,749.47 ± 10,722.43 vs. $32,613.33 ± 38,913.68).

The TSRQ was designed to test for autonomous, controlled and amotivational constructs. Cronbach’s alpha values for motivation for diet were: autonomous: 0.72, controlled: 0.82, amotivational: 0.23. Cronbach’s alpha values for exercise were: autonomous: 0.89, controlled: 0.85, amotivational: 0.35. Removal of single items from the computation for amotivation did not significantly improve alpha (not shown), suggesting too few items or heterogeneous interpretation of the items by this select population of study subjects. Therefore, no further interpretations were made of results pertaining to amotivation. For perceived competence and self-efficacy, Cronbach’s alpha was high (perceived competence for healthy diet: 0.91; perceived competence for exercising regularly: 0.92; self-efficacy: 0.84).

### Motivation, perceived competence, and general self-efficacy

The unadjusted median scores for the TSRQ motivation categories for eating a healthy diet are shown in Table 2. Regarding motivation for eating a healthy diet, there was no difference between groups in scores for autonomous factors. The overweight/obese group demonstrated a significantly higher median score for controlled motivation. Analysis of the 6 controlled items revealed that overweight/obese subjects had significantly higher scores (after Bonferroni correction) for two items: “feel pressure from others to do so” (overweight/obese 2.6 ± 1.9 vs normal weight 2.2 ± 1.3, *p* = 0.0006), and “want others to see I can do it” (5.4 ± 1.9 vs 4.2 ± 2.2, *p* = 0.0001). There were no significant differences in the items “feelings of guilt or shame otherwise,” “others would be upset,” “would feel bad about self,” or “want approval from others.”

**Table 2:** Motivation, perceived competence, and self-efficacy

	Normal weight	Overweight + Obese
	Mean ± SD	Mean ± SD
Motivation for Eating a Healthy Diet		
Autonomous (*n* = 99)	34.1 ± 6.5	36.5 ± 4.6
Controlled (*n* = 96)	18.1 ± 8.1	23.8 ± 9.3**
Motivation for Exercise		
Autonomous (*n* = 99)	33.1 ± 7.9	35.9 ± 6.9
Controlled (*n* = 96)	18.8 ± 8.8	22.6 ± 10.4
Perceived Competence - Diet (*n* = 100) median (25-75% IQR)	20.0 (14.5-24.0)	22.5 (19.0-26.0)*
Perceived Competence - Exercise (*n* = 100) median (25-75% IQR)	23.5 (15.5-25.0)	24.0 (20.5-28.0)
Self-Efficacy (*n* = 77)	31.5 ± 5.4	31.6 ± 4.5

Legend: Subjects completed the Treatment Self Regulation Questionnaire (TSRQ) for eating a healthy diet and exercise, the Perceived Competence Scales for Eating Healthy and Exercising Regularly, and the General Self-Efficacy Scale. Subjects assigned a score between 1 and 7 for each item, with higher scores indicating the statement is more “true” for themselves. The data are expressed as the means ± standard deviation (SD), or as the median with the 25-75% interquartile range (IQR) for non-normal data (perceived competence)

*p < 0.05, **p < 0.01 for comparisons between normal weight subjects and overweight/obese subjects

The scores for the TSRQ on motivation for exercise (Table 2) followed a similar trend, with no significant differences between normal and overweight/obese groups in autonomous scores. Overweight/obese subjects showed a trend toward higher scores for controlled factors (*p* = 0.072).

The perceived competence and general self-efficacy scores are shown in Table 2. For perceived competence for eating a healthy diet, overweight/obese subjects demonstrated higher scores, although no individual item was significantly higher. For perceived competence in exercising regularly, there was no difference in the composite scores.

General self-efficacy was similar between normal weight and overweight/obese subjects.

### The effect of patient characteristics and socioeconomic covariates on controlled motivation and perceived competence for healthy eating in normal vs. overweight/obese subjects

Correlations were performed in the entire cohort to determine whether patient characteristics and socioeconomic factors were related to the TSRQ, PCS, and GSES scores (Table 3). Although paternal education and family income were lower in the overweight/obese group, there were no correlations in the entire cohort between either of these factors and the scores.

**Table 3:** Correlations between motivation, perceived competence, or self-efficacy and select characteristics

	Motivation				Perceived Competence		Self-Efficacy (*n* = 77)
	Diet (*r*)		Exercise (*r*)		Diet	Exercise	
	Autonomous	Controlled	Autonomous	Controlled	(ρ)	(ρ)	(*r*)
Age, years	−0.026	−0.173	0.037	0.053	−0.043	−0.012	0.098
Mother’s highest education level (grade)	−0.118	0.011	−0.050	−0.032	0.063	0.11	0.223
Father’s highest education level (grade)	−0.070	−0.11	−0.096	−0.093	−0.037	−0.0081	0.151
Annual family Income ($) (*n* = 85)	ρ = −0.13	ρ = 0.13	ρ = −0.081	ρ = 0.094	−0.13	−0.047	0.097

Legend: Pearson or Spearman’s correlations were performed between composite scores and patient/family characteristics. Data are represented as Pearson’s *r* or Spearman’s ρ where indicated

Paternal education and family income were then examined separately in different ANCOVA models (Table 4). Age and sex were used as covariates in all models. When controlled motivation was compared between normal and overweight/obese groups, with paternal education as an additional covariate, the scores remained significantly higher among overweight/obese subjects. When annual family income was substituted as the additional covariate, the difference persisted.

**Table 4:** The effect of age, gender, and socioeconomic covariates on controlled motivation and perceived competence for healthy eating

	Normal weight (LSM ± SE)	Overweight + Obese (LSM ± SE)	*p*-value
Controlled motivation for eating a healthy diet			
Unadjusted	17.9 ± 1.5	23.8 ± 1.2	0.002
Age, Gender	18.1 ± 1.5	23.7 ± 1.2	0.004
Age, Gender, Father’s Education	18.7 ± 1.5	24.3 ± 1.3	0.007
Age, Gender, Family Income	18.2 ± 1.6	23.1 ± 1.3	0.02
Perceived competence for eating a healthy diet			
Unadjusted	19.6 ± 0.9	22.0 ± 0.7	0.03
Age, Gender	19.5 ± 0.9	22.1 ± 0.7	0.02
Age, Gender, Father’s Education	19.5 ± 0.9	22.2 ± 0.7	0.02
Age, Gender, Family Income	19.1 ± 0.9	22.0 ± 0.8	0.02

Legend: For controlled motivation, female gender had a positive effect, father’s education had a positive association, while age and family income had negative associations. However, the effects of these covariates were not statistically significant, and controlled motivation remained higher in the overweight/obese group after adjustment. For perceived competence for eating a healthy diet, female gender had a negative effect, while age, father’s education, and family income had positive associations. However, the effects of these covariates were not statistically significant, and perceived competence remained higher in the overweight/obese group after adjustment. Data are represented as the least square means (LSM) and standard error (SE) for each group

Similarly, for perceived competence for eating healthy, overweight/obese subjects continued to demonstrate higher scores with paternal education as the additional covariate. This difference also remained after substituting family income as the additional covariate.

### Exploratory relationships between TSRQ, PCS, or GSES scores and blood pressure or patient self-reported behaviors

We explored whether the TSRQ, PCS, and GSES scores were related to patient systolic blood pressure percentiles or self-reported behaviors through Pearson’s or Spearman’s correlations analyses of the entire cohort. Systolic blood pressure percentiles were positively correlated (*p* < 0.05) to autonomous motivation for diet (correlation coefficient *r* = 0.270), controlled motivation for diet (*r* = 0.257), and autonomous motivation for exercise (*r* = 0.279). Perceived competence for eating healthy was negatively correlated with number of sodas consumed per day (ρ = −0.21, p < 0.05), screen time per day (ρ = −0.29, *p* < 0.01), and fast food per day (ρ = −0.37, p < 0.01).

## Discussion

Assessment of motivation and confidence in ability to initiate and maintain behavior changes is essential in counseling for lifestyle modification in weight management. This study aimed to determine type of motivation, perceived competence, and general self-efficacy among overweight/obese adolescents receiving health care from pediatric ambulatory clinics in South Los Angeles County compared to normal weight adolescents. Our results demonstrated the following points regarding this cohort: 1) overweight/obese adolescents exhibited similar autonomous motivation, but higher controlled motivation for healthy eating in comparison with normal weight adolescents; 2) overweight/obese adolescents demonstrated higher perceived competence in relation to healthy eating; 3) the socioeconomic factors of family income and paternal education did not fully explain the variation in controlled motivation and perceived competence for healthy eating. These findings contribute to the current literature as they provide unique results from a study population of adolescents, including a high proportion of Latinos, at a publicly-funded ambulatory care center in South Los Angeles. Moreover, this study adds to the literature on motivation for healthy eating, which appears to be studied less frequently than motivation for exercise or physical activity.

Our study cohort included a high proportion of subjects who identified as Latino, which is a group that experiences higher rates of obesity (38.9% overweight or obese nationwide) [1]. Comparison of normal weight and overweight/obese adolescents yielded two differences in socioeconomic characteristics: estimated family income and paternal education level. Lower family income in the overweight/obese group is consistent with local data on higher prevalence of obesity observed at lower income levels [24], while lower paternal education level is also consistent with findings from one German study showing a strong inverse relationship between obesity and parental education [35]. Analysis of data from a large, school-based health exam and questionnaire found that paternal education was independently associated with childhood obesity after accounting for other socioeconomic factors.

Regarding exercise, no differences in motivation were observed among overweight/obese adolescents in contrast to a previous study demonstrating greater external regulation and amotivation among obese Korean students [13]. Possible reasons for the contrast may be related to differences in study settings (cited study conducted in Korea), and study populations (Korean students recruited from junior high schools, not those seeking medical care). Regarding eating a healthy diet, our study showed that overweight/obese adolescents exhibited similar autonomous motivation, but greater controlled motivation, in contrast to our hypothesis. As proposed by the Self-Determination Theory, autonomy is an innate desire to be a causal agent of change in one’s own life, and is one of the universal psychological needs. Autonomous motivation is supported in the literature as a predictor of success in weight management [36]. In contrast, achieving behavior change in response to controlled motivation is not as satisfying to the individual, and thus has not been found to be associated with long-term behavior change or weight loss [36, 37]. The difference in the controlled motivation scores was not accounted for by parental education or family income, and therefore additional social factors and influences should be considered.

While external social pressure is not among the most common motivators for weight control among the adult population [38], adolescents are more vulnerable to external influence [39] which may affect motivation. Our study results did not provide evidence for feelings of guilt or shame, or feeling bad about oneself, as the major contributors to controlled motivation. “Feel pressure from others to do it” and “want others to see I can do it” were the statistically significant specific items contributing to higher controlled motivation for eating healthy among overweight/obese subjects. Therefore, the higher controlled motivation may result from receiving feedback from others. This may be illustrated in the example of an adolescent who joins a “weight loss challenge group” with family or friends. The group members agree to make lifestyle changes together, and they compete to see who can lose the most weight. The adolescent therefore feels group pressure to make changes leading to weight loss, and wants to show the group members (s)he can do it. Our results may also be related to social desirability, which is the tendency to behave or respond in a way that is believed to be viewed favorably by others. While we did not measure social desirability directly, it is possible that higher controlled motivation in our cohort is related to wanting to fit in and conforming to social expectations in their peer groups.

Exposure to assessments and interventions from health care providers may also have provided a context for controlled motivation. Since the subjects were recruited from an ambulatory pediatric clinic setting, it is likely that the overweight/obese subjects had already experienced screening and interventions for obesity. Normal weight subjects would have experienced less screening, and fewer interventions. Published evidence on adolescents indicates that both autonomous and controlled motivation can increase during obesity interventions [28]. The statistically significant exploratory association between systolic blood pressure and controlled motivation for eating healthy supports the possibility that overweight/obese subjects were receiving feedback on their eating habits in part due to the presence of a medical condition (hypertension). Therefore, it is possible that past interventions influenced this study’s outcome. Nevertheless, in clinical practice, provider communication efforts should be directed toward understanding the context of controlled motivation and identify barriers to behavior change.

Motivation may mediate perceived competence for healthy behaviors [40]. While there were no differences in perceived competence regarding exercise, overweight/obese adolescents demonstrated higher perceived competence for eating healthy. The exploratory correlations analyzed for potential relationships between the main outcome composite scores and health-related behaviors. The perceived competence scores demonstrated an inverse relationship with the frequency of engagement in unhealthy habits (eating fast food, soda consumption, and screen time). Thus, limiting unhealthy behaviors among this population seems to be associated with confidence in eating healthy. In this respect, we speculate on the possibility that an overweight/obese adolescent with controlled motivation may respond to the external influence of family, friends, or physicians by internalizing their feedback and decreasing unhealthy behaviors. After the change, the adolescent sees that he or she is capable of the change, leading to development of self-confidence in the ability to carry out health-promoting behaviors such as eating healthy.

Limitations of this study included a relatively small sample size, and therefore it is unknown whether the results are influenced by additional factors such as ethnicity. The study is also unable to determine whether the level of parental involvement (e.g. single parents or separated parents) affected outcomes. The cross-sectional study design allowed for examination of associations but not cause-and-effect relationships. Consequently, we are unable to assess for impact of motivation, perceived competence or self-efficacy on potential changes in BMI. Including patients with chronic conditions limits generalizability of results to other populations. The sample size also limits study of subgroups of patients, including those with different types of chronic diseases. Adolescents may also have varying definitions of eating a healthy diet and exercising regularly, however this was a study that focused on patient perceptions.

## Conclusions

Overweight and obese adolescents from a pediatric ambulatory center in South Los Angeles exhibit no difference in autonomous motivation in comparison to normal weight subjects, and demonstrate higher controlled motivation and perceived competence for eating a healthy diet. In the clinical practice of weight management, providers should carefully assess adolescents for type of motivation and perceived competence in order to develop patient-centered care plans. Providers should recognize the potential influence of patients’ families and friends, as well as themselves, in contributing to type of motivation. Each patient’s own motivation and sense of confidence should be integrated into the intervention plan to meet the patient’s health-related goals. Emphasis on autonomous factors during counseling may promote sustainability of health-promoting behaviors in the presence of high controlled motivation. Future studies may examine whether these determinants may predict behavior change or weight loss among these patients in response to interventions.

## Abbreviations

BMI: Body mass index
GSES: General Self-Efficacy Scale
PCS: Perceived Competence Scale
SDT: Self Determination Theory
SES: Socioeconomic status
TSRQ: Treatment Self-Regulation Questionnaire
